# Some New Forms of Orthodontic Mechanism

**Published:** 1917-01

**Authors:** H. C. Pollock


### SOME NEW FORMS OF ORTHODONTIC MECHANISM.

Dr. E. H. Angle (*Dental Cosmos,* September, 1916), announces a new form of appliance and in this paper the difficulties involved in the use of the Angle Pin Tube Appliance are taken up. The need and demand for a modification of the original pin tube appliance is pointed out and a detailed description is given of the appliance, which it is believed will meet the requirements in which the pin tube appliance is lacking. The subject of injudicious extraction of teeth is also discussed and the error of this former practice is called to mind. This discussion leads up to the establishing of normal function and correlated parts. He maintains that "On this basis only can orthodontic treatment be permanently beneficial and truly satisfactory for such only as in accordance with nature, and this is the true meaning of orthodontia."

Leading up to the appliance, however, other subjects are taken up which establish the principle upon which the appliance is based. Indications for early treatment of malocclusion is urged, although a warning is issued against the needless interference with child dentures now so often practiced.

As to the physiologic application of force, the modern idea is presented as advocated in previous work by Noyes and Oppenheim as being the ideal force to be desired, and with this end in view, the improved appliance should operate in such a physiologic way as to meet such requirements. They should move teeth slowly, by slow, gentle force continuously applied which will not only move teeth far more rapidly than the great force, but does not inhibit the normal cellular activity as has been demonstrated to be true in using great force.

After describing in detail the technique of the new appliance, the advantages of the new devise are summed up as follows:

"Force may be so controlled as to permit or to prevent the tipping of any tooth or teeth to any extent, or to compel the bodily movement of any tooth or teeth in either or both arches.

"This mechanism is of the greatest simplicity, of the maximum delicacy of parts, and with all unnecessary material eliminated. Hence it is of the least inconvenience to patients and the easiest to keep cleansed. It would seem that the mechanism is nearly ideal, not only for securing the necessary static force for anchorage and of dynamic force for tooth movement, but for directing and controlling this force so that all cellular change attending on tooth movement most nearly accords with the laws of physiology. It is also graceful in its proportions and not unpleasing in appearance. In a word, the principles of mechanics, art, and physiology do not conflict, but are made to harmonize beautifully and as was never possible in orthodontic mechanism before. It is so simple and easy to apply as greatly to lessen the usual work of the orthodontist and the usual number of visits of patients. It is not expected that it will wholly supersede the pin and tube mechanism, neither will it wholly supplant the expansion arch in its round form with ligature attachments. In fact, the ligature attachment will be found to be of advantage in connection with the ribbon arch in the movement of premolars and of other teeth that may be so pronouncedly misplaced as to render impracticable the bending of the ribbon arch to gain bracket attachment with them until after they have first been moved into more favorable positions by means of ligatures. But in the great majority of cases the mechanism herein shown will be found to possess such obvious advantages in force, control and in ease of application and operation, that I believe it will find a permanent place in orthodontia."

A few cases are shown which according to the author have been successfully treated by Ketcham, Gough, Strang, and Mendell, as well as those of the author.—H. C. Pollock, in *International Journal of Orthodontia.*

